# *ACE2* and *TMPRSS2* SNPs as Determinants of Susceptibility to, and Severity of, a COVID-19 Infection

**DOI:** 10.3389/bjbs.2021.10238

**Published:** 2022-01-12

**Authors:** S. Abdelsattar, Z. A. Kasemy, S. F. Ewida, R. A. A. Abo-Elsoud, A. A. Zytoon, G. A. Abdelaal, A. S. Abdelgawad, F. O. Khalil, H. F. M. Kamel

**Affiliations:** ^1^ Department of Clinical Biochemistry and Molecular Diagnostics, National Liver Institute, Menoufia University, Menoufia, Egypt; ^2^ Department of Public Health and Community Medicine, Faculty of Medicine, Menoufia University, Menoufia, Egypt; ^3^ Department of Medical Physiology, Faculty of Medicine, Menoufia University, Menoufia, Egypt; ^4^ Department of Radiodiagnosis, Faculty of Medicine, Menoufia University, Menoufia, Egypt; ^5^ Department of Chest Diseases and Tuberculosis, Faculty of Medicine, Menoufia University, Menoufia, Egypt; ^6^ Department of Clinical Pathology, National Liver Institute, Menoufia University, Menoufia, Egypt; ^7^ Department of Clinical Microbiology and Immunology, National Liver Institute, Menoufia University, Menoufia, Egypt; ^8^ Department of Biochemistry, Faculty of Medicine, Umm Aloura University, Makka, Saudi Arabia; ^9^ Department of Medical Biochemistry and Molecular Biology, Faculty of Medicine, Ain Shams University, Cairo, Egypt

**Keywords:** ACE2 gene, TMPRSS2 gene, thromboembolic complications, determinants of COVID-19 disease, cytokine storm, genetic susceptibility of COVID-19

## Abstract

**Background:** Genetic risk factors may be related to the infectivity and severity of SARS-CoV-2 infection. Angiotensin-converting enzyme 2 (ACE2) and host transmembrane serine protease (TMPRSS2) have key role in viral cell entrance and priming.

**Methods:** This case-control study on 147 healthy controls and 299 COVID-19 patients identified potential determinants and risk factors, including gene polymorphism involved in the severity (mild, moderate, severe) of COVID-19 disease defined by CORAD radiological criteria.

**Results:** The ACE2 s2285666 and TMPRSS2 rs12329760 SNPs were significantly linked with COVID-19 disease severity, as were certain co-morbidities (hypertension, heart disease) and laboratory parameters. Both SNPs were amongst the highest predictors of disease severity: TMPRSS2 rs12329760 CT + TT [odds ratio (95% CI) 17.6 (5.1–61.10), ACE2 rs2285666 CT + TT 9.9 (3.2–30.9), both *p* < 0.001]. There was an increase in the expression of genotype frequencies of ACE2 rs2285666 and TMPRSS2 rs1232976 (TT), (CT + TT), and (T) allele in severe COVID-19 group compared to control and mild groups. Disease severity was also linked to elevated CRP, ferritin and D-dimer, and lower lymphocytes and platelet count (all *p* < 0.001).

**Conclusion:** ACE2 rs2285666 and TMPRSS2 rs12329760 SNPs, in addition to lymphocyte count, CRP, D-dimers, ferritin, and hypertension, are predictors of COVID-19 disease severity.

## Introduction

The existing pandemic of the coronavirus disease 2019 (COVID-19) has produced universal crisis, devastating health organizations (1). It is caused by the severe acute respiratory syndrome coronavirus 2 (SARS-CoV-2), a new beta coronavirus sharing 79% sequence similarity with SARS-CoV, the agent which provoked the 2003 SARS outbreak (2). Unlike other CoVs, SARS-CoV-2 was found to have a greater worldwide spread thus it has infected more individuals than SARS-CoV-1 and the Middle East respiratory syndrome (MERS-CoV) combined (3).

COVID-19 dramatically increases morbidity and mortality with existing risk factors such as age and co-morbidities. Whereas most of the infected persons recover, even young and unpredictably, healthy subjects may be succumbed to the illness (4). These observations have raised the question of how the variation in COVID-19 disease severity could be explained by genetic susceptibility. The genetic factors might be related to highest extreme transmission of SARS-CoV-2 and the observed progression of the disease in a considerable proportional of the diseased population; so far, these factors are mostly undetermined. Extensive genetic studies in various populations from different geographic origins have exhibited considerable genetic disparity in protein-coding areas, with broadly variable allelic’ frequencies (5).

**FIGURE 1:**
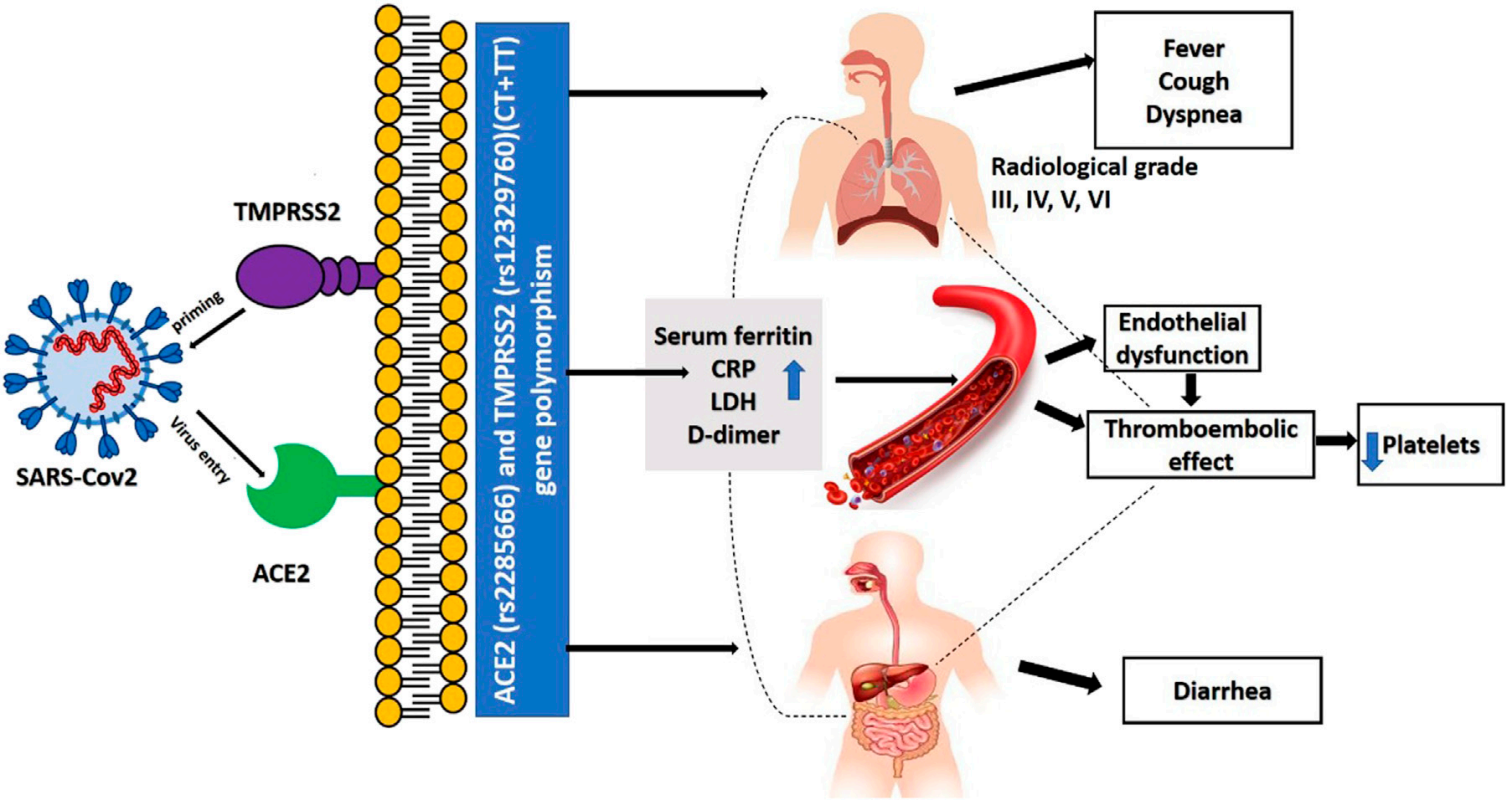
Role of ACE2 and TMPRSS2 in the pathogenesis of covid-19 infection.

SARS-CoV-2 contagion is dependent mainly on the cellular factors of the infected host. Angiotensin-converting enzyme 2 (ACE2) allows entrance, and the transmembrane serine protease (TMPRSS2) permits SARS-CoV-2 spike (S) protein priming (6). ACE2, encoded at Xp22.2 (7), is a metallopeptidase that catalyzes conversion of angiotensin II into angiotensin I (8). TMPRSS2, encoded at 21q22.3, whose transcriptional activity is controlled by androgen receptors and which has roles in carcinogenesis (9,10). It also has specific role in cleaving viral S glycoprotein and consequently facilitating viral activation, thus it is considered one of the crucial factors for pathogenesis of SARS-CoV-2 (6), and inhibitors of serine proteases of TMPRSS2 block SARS-CoV-2 pathogenicity (6). TMPRSS2 also has an essential role in the cleavage of arginine and lysine residues of ACE2, thus augmenting the role of ACE during viral entry and uptake into the host cell (11) (as shown in Figure 1).

The deterioration from mild to severe form of COVID-19 has raised the question of whether genetic factors can determine the susceptibility and/or severity of symptoms, in addition to an exaggerated immunological responses to the virus which commonly leads to a poorer outcome (12). Features involved in the spread of SARS-CoV-2 include its molecular structure, ethnic prevalence and longevity of their immune response, disease pathophysiological consequences, therapeutic interventions, and vaccination (13). Since there is considerable variation in disease behavior among COVID-19 infected patients; a multifactorial analysis may identify the possible risk factors that is involved in development and progression of COVID-19 infection. Whilst the greater part of our knowledge of the epidemiology and pathogenesis of COVID-19 has arisen from Chinese populations, relatively little in known in other racial groups. We therefore hypothesised roles for ACE2 (rs2285666) and TMPRSS2 (rs12329760) SNPs for prediction of susceptibility and severity of COVID-19 infection concerning clinical findings, laboratory investigations, and radiological data in a mixed Caucasian and African population.

## Patients and Methods

We tested our hypothesis on 147 healthy controls and 299 COVID-19 patients enrolled between December 2019 and October 2020. Informed written consent was obtained before volunteers’ or patients’ enrollment. This study was approved by National Liver disease Institute review board (NLI IRB protocol N. 00222/2020). Inclusion criteria for the COVID-19 patients’ group involved patients with positive Reverse Transcription polymerase chain reaction (RT-PCR) for SARS-COV-2. Negative RT-PCR patients and patients with a proved other concurrent acute illness were excluded from the study. At the same time, control subjects were confirmed to be negative by RT-PCR for COVID-19.

Clinical data including age, sex, high risk occupations (physicians, nurses, pharmacists or social workers in hospitals or health care centers who are in close contact with patients, drivers, security, delivery and restaurant workers, cleaners, and teachers), co-morbidity (hypertension, diabetes mellitus, heart disease, bronchial asthma) and symptoms (fever, cough, sore throat, muscle ache, headache, dyspnea, diarrhea, loss of taste, loss of smell). Diagnosis was confirmed by SARS-CoV-2 RT-PCR test of nasopharyngeal or oropharyngeal swabs collected in sterile tubes, each containing 5 ml viral transport media according to World Health Organization (WHO) recommendations (4°C for duration around 5 days and −70°C for longer duration than 5 days) using the genesig Real-Time PCR Coronavirus (COVID-19) CE IVD assay (14) which is an *in vitro* diagnostic test based on Real-Time PCR technology (QIAGEN, GmbH), developed for specific detection of SARS-CoV-2 viral RNA.

All patients underwent chest high-resolution computed tomography (HRCT). A single breath-hold with full respiration was used for examination. CT scoring was performed by clinical-data-blinded radiologists. Patients’ classification and grouping into mild, moderate, and severe were done using a CORAD radiologic scoring system previously described by Bai et al. (15) CORADS 1; normal CT, COVID-19 is highly unlikely. CORADS 2; signs of bronchiolitis as other infections with low level of COVID-19 infection suspicion. CORADS 3; Bronchopneumonia and ground-glass opacity, which is indeterminate for COVID-19 infection. CORADS 4; Unilateral ground glass with multifocal consolidations, which are highly suspicious for COVID-19 infection. CORADS 5; multifocal ground-glass appearance with consolidation. CORAD 6; positive PCR with ground-glass appearance bilaterally. Under these criteria, the most common CT features were pulmonary ground glass opacity in 91.9% with bilateral and diffuse distribution in 91 and 69.8% of the patients, respectively, and air space consolidation in 64.2% of the patients. Smooth interlobular septal thickening 64.9%. Pleural thickening and effusion and mediastinal lymphadenopathy were reported in 16, 15, and 30% of the patients, respectively**.**

Blood Sampling and methodologies were as follows. Ten ml of venous blood were withdrawn from the cubital vein of each subject in the study divided as follow: 3 ml were transported slowly into vacutainer EDTA tube frozen at −20°C for consequent DNA extraction and genotyping, and 2 ml of blood were transferred into another EDTA tube for determination of CBC by Sysmex XT-1800i automated hematology analyzer (Sysmex, Japan). Three ml were transferred into a plain tube for centrifugation and collection of serum for subsequent biochemical analysis of CRP by particle enhanced immunoturbidimetric assay using Cobas c501 Auto analyzer (Roche-Germany), Ferritin and LDH by particle enhanced immunoturbidimetric assay using Cobas e 601 Auto analyzers (Roche-Germany). The last 2 ml of blood were collected in sodium citrate (3.2%) vacutainer tubes for D. dimer determination using (D-DI2 Tina-quant D-Dimer Gen.2) on the Cobas 6000 analyzer (c501 module) with an expected value less than 0.5 µg fibrinogen equivalent/ml (ug FEU/ml) (16).

Genotyping of ACE2 (rs2285666) and TMPRSS2 (rs12329760) genes polymorphisms were as follows. Genomic DNA was extracted from all samples using Gene JET TM whole blood Genomic DNA purification Mini kit (Thermo Scientific EU/Lithuania) according to the manufacturer’s instructions. DNA concentration of each sample was assessed using a Nanodrop spectrophotometer (UV spectrophotometer Q3000, Quawell Technology, Inc., United States). ACE2 (rs2285666) and TMPRSS2 (rs12329760) SNPs were analyzed using real-time polymerase chain allelic discrimination technology using TaqMan SNP genotyping assay kit (Thermo Fisher Scientific, Waltham, MA, United States) has been previously described (17), with catalog no C___2551626_1_&C__25622353_20 and their context sequences as follow respectively: (VIC/FAM) ATA​ATC​ACT​ACT​AAA​AAT​TAG​TAG​C(C/T)TAC​CTG​GTT​CAA​GTA​ATA​AGC​ATT​C. (VIC/FAM) CAG​GAC​TTC​CTC​TGA​GAT​GAG​TAC​A(C/T)CTG​AAG​GAT​GAA​GTT​TGG​TCC​GTA​G. The PCR reaction mixture contained 7.5 μl of TaqMan Genotyping master mixture (Applied Biosystems), 0.75 μl of TaqMan SNP (probes), 1 μl of genomic DNA (1–10 ng), and 5.75 μl nuclease-free water. Thermal conditions were as follows: initial denaturation at 95°C for 10 min, 40 cycles were run at 95°C for 15 s (denaturing) followed by 60°C for 1 min (annealing/extension). PCR amplification was performed by Rotor gene Q Real-Time PCR System reaction (QIAGEN, GmbH-Germany) in duplicates with 100% concordance.

Analyses were performed using SPSS version 22.0 (SPSS Inc., Chicago, IL, United States). Pearson’s Chi-square (χ^2^) test was used to determine significance of the association between two categories. Linear-by-Linear Association (LLA) test for trend results of the Crosstab procedure was applied, and it equals (N-1)*r^2, where N is the sample size, and r is the Pearson correlation between the 2 variables. LLA test, also known as the Mantel-Haenzsel test for trend. Cramér’s V was used to detect the presence of trends in a series of data by estimating the effect size following Linear-by-Linear Association. Shapiro-Wilk test as one of the normality tests was conducted to ascertain the normality of distribution, and accordingly, Unpaired t-test was used for parametric data while Mann-Whitney test was used for non-parametric ones. Linear trend analysis using the Jonckheere-Terpstra test was applied to detect whether there was an increasing or decreasing trend across the ordered groups. Mann-Kendall (M-K) test is used to detect the presence of linear or non-linear trends (steadily increasing/decreasing or unchanging) in a series of data by estimating the effect size following Jonckheere-Terpstra (J-T) Test. The two ACE2 (rs2285666) and TMPRSS2 (rs12329760) were tested for Hardy Weinberg equilibrium (HWE) in patients and controls. For additional analysis of the association between ACE2 (rs2285666) and TMPRSS2 (rs12329760), genes polymorphisms, and disease severity, 95% confidence interval (95% CI), and the odds ratio (OR) were consequentially calculated in dominant, recessive1 and 2, co‐dominant, and over‐dominant genetic models. Binary logistic regression analysis was conducted to detect the independent predictors for disease severity. Multiple comparisons were tested using Holm-Bonferroni Sequential Correction: An EXCEL Calculator ^©^ Justin Gaetano, 2013. *p*-values are statistically significant after this correction.

## Results

Table 1 shows clinical and occupation data, co-morbidities, and symptoms. Almost all were linked to disease severity. The greatest six effect sizes were (in order) dysponea, muscle ache, headache, hypertension, fever, and diarrhea. Table 2 shows laboratory data, many of which were significantly linked to disease severity. The greatest six effect sizes were (in order) CRP, ferritin, D-dimers, platelets, lymphocytes, and the neutrophil to lymphocyte ratio. Hardy-Weinberg Equilibrium (HWE) Genotype Frequencies of ACE2 (rs2285666) were significantly out of equilibrium in all diseased groups (all *p* < 0.001), but the TMPRSS2 (rs12329760) was out of equilibrium only in the group with severe symptoms (*p* = 0.009).

**TABLE 1:** Demographic criteria and COVID-19 symptoms of the studied groups.

	Controls (n = 147)		Disease severity						Effect size (CI 95%)	*p* Value
			Mild (no = 116)		Moderate (n = 128)		Severe (n = 55)			
	No	%	No	%	No	%	No	%		
Age (y) $\overset{¯}{x}$ ±SD	45.7 ± 14.7		41.9 ± 15.0		50.2 ± 16.6		58.0 ± 14.7		0.17 (0.10–0.24)	<0.001
Sex										
Male	75	51.0	47	40.5	74	57.8	31	56.4	0.13 (0.06–0.23)	0.044
Female	72	49.0	69	59.5	54	42.2	24	43.6		
Occupation										
Risky	26	17.7	42	36.2	49	38.3	37	67.3	0.32 (0.24–0.42)	<0.001
Not risky	121	82.3	174	63.8	79	61.7	18	32.7		
Co-morbidity										
Hypertension	-	-	9	7.8	43	33.6	28	50.9	0.36 (0.28–0.46)	<0.001
Diabetes Mellitus			15	12.9	30	23.4	15	27.3	0.15 (0.05–0.26)	0.015
Heart disease			6	5.2	21	16.4	11	20.0	0.19 (0.10–0.28)	0.002
Bronchial asthma			4	3.4	7	5.5	10	18.2	0.21 (0.08–0.36)	0.001
Symptoms										
Fever	-	-	73	62.9	116	90.6	51	92.7	0.35 (0.24–0.46)	<0.001
Cough			95	81.9	100	78.1	53	96.4	0.18 (0.11–0.26)	0.070
Sore throat			38	32.8	30	23.4	17	30.9	0.09 (0.03–0.22)	0.520
Muscle ache			22	19.0	80	62.5	36	65.5	0.43 (0.33–0.53)	<0.001
Headache			31	26.7	79	61.7	44	80.0	0.42 (0.31–0.51)	<0.001
Dyspnea			5	4.3	67	52.3	40	93.0	0.57 (0.48–0.64)	<0.001
Diarrhea			12	10.3	16	12.5	17	30.9	0.21 (0.09–0.34)	0.002
Loss of taste			47	40.5	40	31.3	9	16.4	0.18 (0.09–0.29)	0.022
Loss of smell			69	59.6	51	39.8	25	45.5	0.18 (0.08–0.30)	0.002

Data were presented as $\overset{¯}{x}$ ± SD or no, %. Linear-by-Linear Association (LLA) test for trend results was applied for qualitative data. Cramér’s V was used to estimate the effect size following Linear-by-Linear Association**.**

**TABLE 2:** Laboratory data of the studied groups.

	Controls (n = 147)	Disease severity			J-T	Effect size (CI 95%)	*p* Value
		Mild (no = 116)	Moderate (n = 128)	Severe (n = 55)			
Hb(g/l)	127 ± 18	125 ± 15	128 ± 20	119 ± 19	0.83	−0.03 (−0.10 to 0.04)	0.403
RBCs*10^6^	5.0 ± 0.4	5.0 ± 1.6	5.1 ± 1.9	4.7 ± 1.8	1.73	−0.15 (−0.22) to (−0.07)	<0.001
HCT%	42.1 ± 3.7	38.3 ± 6.0	38.2 ± 8.1	36.8 ± 8.3	0.08	−0.16 (−0.23) to (− 0.09)	<0.001
Platelets*10^3^	322 (257–371)	209.5 (176–277.5)	215 (162–248.7)	188 (120–245)	2.87	−0.38 (−0.44) to (−0.32)	<0.001
WBCs*10^3^	6.3 (4.9–7.1)	5.9 (4.8–7.2)	5.7 (3.6–7.5)	5.5 (3.4–10.6)	0.77	−0.04 (−0.09) to ( 0.04)	0.232
Lymphocytes*10^3^	1.7 (1.5–2.4)	1.5 (1.1–2.1)	1.1 (0.6–1.6)	0.8 (0.5–1.1)	9.39	−0.35 (−0.41) to (−0.29)	<0.001
Neutrophils*10^3^	3.7 (3.0–4.6)	3.7 (2.8–5.8)	2.5 (1.5–3.1)	3.1 (2.2–7.6)	5.85	−0.18 (−0.25) to (−0.11)	<0.001
NLR	2.1 (1.6–2.6)	2.6 (1.6–3.7)	2.3 (1.5–3.5)	3.9 (2.6–8.8)	3.08	0.19 (0.13) to (0.26)	<0.001
PLR	10.2 (8.5–12.7)	9.4 (5.3–13.5)	10.7 (6.3–15.10	12.2 (7.6–20.1)	3.18	0.06 (−0.001) to (0.13)	0.077
CRP (mg/L)	2.3 (1.4–3.5)	13.8 (6.8–28)	70 (25.7–190)	114 (82.9–150.20	11.19	0.70 (0.67–0.72)	<0.001
Ferritin (mg/L)	17 (12–25)	120 (61.3–199)	257.5 (132–645.5)	612.0 (345.6–831)	9.26	0.67 (0.63–0.70)	<0.001
LDH (mg/L)	285 (248–321)	336.5 (250–388.5)	334.5 (250.8–600)	381.0 (280–776)	2.47	0.22 (0.15–0.28)	<0.001
D-Dimer (mg/L)	100 (100–180)	245 (140–530)	310 (140–786.3)	430 (200–1210)	2.74	0.38 (0.32–0.44)	<0.001

Data are expressed as Mean ± SD or Median (Interquartile range). Linear trend analysis using the Jonckheere-Terpstra test was applied to detect whether there was an increasing or decreasing trend across the ordered groups. Effect size was estimated using Mann-Kendall (M-K) test to detect the presence of linear or non-linear trends (steadily increasing/decreasing or unchanging) in a series of data following Jonckheere-Terpstra (J-T) Test.

CI 95%, Confidence interval at 95%; Hb, Hemoglobin; RBCs, Red blood cells; HCT, Hematocrit; WBCs, white blood cells, lymphocytes and neutrophils were presented in absolute figures; NLR, Neutrophil lymphocyte ratio; PLR, Platelets lymphocyte ratio; CRP, C- reactive protein; LDH, lactate dehydrogenase.

Table 3 shows odd’s ratios (OR) and 95% confidence intervals (CI) for the genotypes and alleles of each gene. Regarding the ACE2 SNP, both were no difference between controls and those with mild symptoms, but the TT genotype and T allele was a strong predictor of moderate disease, whilst the CT and TT genotypes, and the T allele, all strongly predicted severe disease. In contract, the TMPRSS2 CC and TT. Table 4 shows recessive, dominant, co-dominant and over-dominant models for genotype combinations. The CC v T, CC + CT v TT and CC v CT + TT models of the ACE2 SNP were all significant predictor of COVID-19 infection, whilst all models of the TMPRSS2 SNP were significant predictors.

**TABLE 3:** ACE2 (rs2285666) and TMPRSS2 (rs12329760) gene polymorphisms in patients’ groups and the controls.

Variables	Control		Mild COVID-19		OR (CI 95%)	Moderate COVID-19		OR (CI 95%)	Severe COVID-19		OR (CI 95%)
	No	%	No	%		No	%		No	%	
ACE2 (rs2285666)											
CC	114	77.6	91	78.4	1.0 (Ref.)	74	57.8	1.0	31	56.4	1.0
CT	21	14.3	12	10.3	0.72 (0.33–1.53)	0	0.0	-	10	18.2	1.75 (0.75–4.10)*
TT	12	8.2	13	11.2	1.36 (0.59–3.12)	54	42.2	6.93 (3.48–13.83)*	14	25.5	4.29 (1.80–10.21)*
Alleles											
C	249	84.7	194	83.6	1.0 (Ref.)	148	57.8	1.0	72	65.5	1.0
T	45	13.3	38	16.4	1.08 (0.68–1.74)	108	42.2	4.04 (2.70–6.04)*	38	34.5	2.92 (1.76–4.84)*
TMPRSS2 (rs12329760)											
CC	123	83.7	98	84.5	1.0 (Ref.)	107	83.6	1.0	31	56.4	1.0
CT	21	14.3	15	12.9	0.90 (0.44–1.83)	21	16.4	1.15 (0.60–2.22)	15	27.3	2.83 (1.31–6.13)*
TT	3	2.0	3	2.6	1.26 (0.25–6.36)	0	0.0	-	9	16.4	11.90 (3.04–46.60)
Alleles											
C	267	90.8	211	90.9	1.0 (Ref.)	235	91.8	1.0	77	70.0	1.0
T	27	9.2	21	9.1	0.98 (0.54–1.79)	21	8.2	0.88 (0.49–1.60)	33	30.0	4.24 (2.40–7.48)*

*p < 0.05.

OR, Odd’s Ratio; CI, confidence interval; ACE2, Angiotensin-converting enzyme 2; TMPRSS2, Transmembrane serine protease 2; C, cytosine; T, thymine; Ref, Reference.

**TABLE 4:** Distribution of ACE2 and TMPRSS2 SNPs in different genetic models.

	OR (95% CI)	*p* value
ACE2 (rs2285666)		
CC vs. TT (Recessive-1)	10.83 (5.36–20.71)	<0.001
CC + CT vs. TT (Recessive-2)	10.15 (5.34–19.30)	<0.001
CC vs. CT + TT (Dominant)	5.01 (3.18–7.89)	<0.001
CC vs CT (Co–dominant)	1.68 (0.89–3.19)	0.108
CT vs. CC + TT (over dominant)	0.94 (0.50–1.76)	0.848
TMPRSS2 (rs12329760)		
CC vs. TT (Recessive-1)	5.03 (1.40–18.14)	0.007
CC + CT vs. TT (Recessive-2)	4.21 (1.17–15.13)	0.017
CC vs. CT + TT (Dominant)	3.30 (1.97–5.52)	<0.001
CC vs CT (Co–dominant)	3.05 (1.76–5.29)	<0.001
CT vs. CC + TT (over dominant)	0.35 (0.20–0.60)	<0.001

Genotype on the left is the reference.

OR, Odds ratio; CI 95%, Confidence interval at 95%; ACE2, Angiotensin-converting enzyme 2; TMPRSS2, Transmembrane serine protease 2; C, cytosine; T, thymine.

Table 5 shows results of a binary logistic regression analysis performed to determine those factors mostly likely to predict disease severity. It was found that disease severity was associated with age, TMPRSS2 variant CT + TT, ACE2 variant CT + TT, neutrophils, ferritin level and CRP. The logistic regression model was statistically significant, χ = 90.55 *p* < 0.001. The model explained 86% (Nagelkerke R2) of the variance in severity and correctly classified 94.8% of cases. Table 6 shows interactions between these genotypes and key laboratory indices. Ferritin, LDH and D-Dimers were strongly linked to the variants in both genes, but the platelet count was strongly linked only to the TMPRSS2 genotype. Similarly, Table 7 shows distribution of symptoms according to genotype. Both were linked strongly to fever, headache, and dysponea, whilst the ACE2 SNP was strongly linked to sore throat and muscle ache, and the TMPRSS2 SNP to diarrhea.

**TABLE 5:** Binary Logistic regression analysis for predictors of COVID-19 infection severity.

	OR (95% CI)	*p* Value
TMPRSS2 (rs12329760) (CT + TT)	17.65 (5.1–61.1)	<0.001
ACE2 (rs2285666) (CT + TT)	9.9 (3.2–30.9)	<0.001
Neutrophils	1.12 (1.07–1.18)	<0.001
Ferritin	0.99 (0.98–1.0)	<0.001
CRP	0.97 (0.95–0.99)	0.002
Lymphocytes	1.09 (1.03–1.16)	0.003
Comorbidity	4.85 (1.63–14.36)	0.004
D-Dimer	0.99 (0.98–1.0)	0.007
Platelets	1.01 (1.0–1.01)	0.024
Male sex	0.54 (0.21–1.36)	0.193
High risk occupation	1.61 (0.61–4.24)	0.331
LDH	1.0 (0.99–1.01)	0.339
WBCs	0.98 (0.93–1.13)	0.720
Age	1.0 (0.97–1.03)	0.757

OR, Odds ratio; CI 95%, Confidence interval at 95%; WBCs, white blood cells; CRP, C- reactive protein; LDH, lactate dehydrogenase; ACE2, Angiotensin converting enzyme 2; TMPRSS2, Transmembrane serine protease 2; C, cytosine; T, thymine.

**TABLE 6:** Distribution of Laboratory data according to ACE2 and TMPRSS2 SNPs.

	ACE2 (rs2285666)		*p* Value	TMPRSS2 (rs12329760)		*p* Value
	CC (no = 196)	CT + TT (n = 103)		CC (no = 236)	CT + TT (n = 63)	
Hb (gm/dl)	12.4 ± 1.8	12.7 ± 1.9	0.124	12.5 ± 1.8	12.8 ± 2.04	0.214
RBCs*10^6^	5.0 ± 1.8	5.1 ± 1.7	0.795	5.1 ± 1.8	4.9 ± 1.7	0.602
HCT%	37.9 ± 7.1	38.2 ± 8.1	0.771	37.9 ± 7.5	38.5 ± 7.1	0.531
Platelets*10^3^	209.5 (172.2–250)	190 (161–250)	0.034	214 (170–256.5)	175 (153–230)	0.001
WBCs*10^3^	5.6 (4.2–7.1)	6.4 (4.7–8)	0.020	5.9 (4.4–7.7)	5.5 (3.8–7)	0.149
Lymphocytes	1141.8 (708–1820)	1137 (708.8–1137)	0.877	1146.9 (716.4–1752)	1083 (638.4–1700)	0.165
Neutrophils	3053 (2178–4697)	2897.5 (1953–1243.6)	0.414	3019 (2061.5–4434.5)	2808 (2025–4031)	0.351
CRP (mg/L)	34.5 (12–96)	66 (18–130)	0.048	42.5 (12–95.8)	73 (24–150)	0.031
Ferritin	172 (100–310)	555 (192–877)	<0.001	190 (103–414)	645.5 (148–900)	<0.001
LDH	324 (250–381)	460 (261–776)	<0.001	342 (250–430)	381 (274–800)	0.004
D-Dimer	200 (100–445)	1100 (320–1450)	<0.001	250 (140–552.5)	1190 (200–1750)	<0.001

Data are expressed as no, %, Mean ± SD or Median (Interquartile range).

ACE2, Angiotensin converting enzyme 2; TMPRSS2, Transmembrane serine protease 2; C, cytosine; T, thymine; Hb, Hemoglobin; RBCs, Red blood cells,; HCT, Hematocrit; WBCs, white blood cells, lymphocytes and neutrophils were presented in absolute figures; NLR, Neutrophil lymphocyte ratio; PLR, Platelets lymphocyte ratio; CRP, C- reactive protein; LDH, lactate dehydrogenase.

Unpaired t test was used for parametric data while Mann-Whitney was used for non-parametric data.

**TABLE 7:** Distribution of associated symptoms according to ACE2 and TMPRSS2 SNPs.

Symptoms	ACE2 (rs2285666)						*p* Value	TMPRSS2 (rs12329760)					*p* Value
	CC (no = 196)			CT + TT (n = 103)				CC (no = 236)			CT + TT (n = 63)		
	No		%	No		%		No	%		No	%	
Fever	148	75.5		92	89.3		0.004	179		75.8	61	96.8	<0.001
Cough	156	79.6		92	89.3		0.034	189		80.1	59	93.7	0.011
Sore throat	33	16.8		52	50.5		<0.001	62		26.3	23	36.5	0.110
Muscle ache	79	40.3		59	57.3		0.005	104		44.1	34	54.0	0.161
Headache	87	44.4		67	65.0		0.001	110		46.6	44	69.8	0.001
Dyspnea	55	28.1		57	55.3		<0.001	78		33.1	34	54.0	0.002
Diarrhea	32	16.3		13	12.6		0.395	26		11.0	19	30.2	<0.001
Loss of taste	60	30.6		36	35.0		0.445	71		30.1	25	39.7	0.147
Loss of smell	94	48.0		51	49.5		0.798	116		49.2	29	46.0	0.660

Data are expressed as no, %, Chi-square test (χ^2^) was used as a test of significance.

ACE2, Angiotensin converting enzyme 2; TMPRSS2, Transmembrane serine protease 2; C, cytosine; T, thymine.

## Discussion

The principle finding in our study was an increase in expression of genotype frequencies ACE2 rs2285666 (TT), (CT + TT), and the allele (T) frequency in moderate and severe COVID-19 groups, and these SNPs were the highest predictors of disease severity by logistic regression analysis. Incorporating these into a model with age, sex, neutrophil count, ferritin, and CRP explained 86% of the variability in disease severity and correctly identified almost 95% of cases.

Our data adds to considerable literature on ACE2 and TMPRSS2 in COVID-19 pathogenesis. For example, the CC or CT genotype causes higher levels of circulating ACE2 in comparison to TT genotype(18), ACE2 lowers blood pressure by several mechanisms as vasodilation; stimulation of sodium and water elimination in urine as well as nitric oxide production (19), and ACE2 levels contribute to SARS-CoV-2 infection severity and complications (20). It has long been established that ACE genotypes are linked to hypertension and so cardiovascular disease (21–27) and we show that hypertension is a leading risk factor for disease severity. We speculate that this is because of a pathophysiological causal link between ACE2 rs2285666, hypertension and disease severity.

The association of elevated D-dimer and COVID-19 disease severity in this study reflects the ongoing activation of the hemostatic system and, potentially, cardiovascular complications. This may be linked to a genetic basis as the increased D-dimer were also significantly associated with the ACE2 rs2285666 (CT + TT) SNP as this genotype is linked to potentially thrombotic atherosclerotic cardiovascular disease (28,29). This prompted Srivastava et al. (30) to speculate that this gene might be a predisposing factor for COVID-19 case-fatality. The significant platelet reduction in the current study was amongst the highest predictors of disease severity and was significantly associated with the expression of ACE2 rs2285666 and TMPRESS2 rs12329760 (CT + TT) gene polymorphism. Thrombocytopenia in COVID-19 infection may be due to platelet aggregation in microthrombus, high autoantibodies increasing platelet destruction, or cytokine storm inducing bone marrow suppression (31).

Several studies have reported increased markers of inflammation, such as CRP, LDH, and ferritin, in COVID-19 infection (13). Our data showed that ferritin, CRP, D-dimers, alongside the lymphocyte and neutrophil counts, contributed to COVID-19 disease severity. Many of these changes may reflect increased cytokine activity, whilst Badawi (32) reviewed the evidence that genetic polymorphisms within an individual’s ACE2-positive cells population is linked to hypercytokinaemia and so their response to COVID-19 disease. Our data fits with this hypothesis, in that we show that ACE2 rs2285666 (CT + TT) SNPs are linked to alterations in inflammatory and other biomarkers (CRP, LDH, ferritin, D-dimer, lymphocytes and neutrophils), some of which are predictive criteria of a COVID-19 cytokine storm (33).

Genotype frequencies TMPRSS2 (rs12329760) TT and CT + TT, and allele frequency T showed a significant increase in the severe COVID-19 group in the current study. Additionally, it was among the highest predictors of disease severity in logistic regression analysis, an observation that supports the hypotheses of Strope et al. (34) and Singh et al. (35) that these SNPs are directly pathogenic. The latter also suggest that sex may have a role in the pathogenicity of the TMPRSS2 SNPs, citing differences in gene responses to androgens, so being linked to sex polymorphism-specific effects, as TMPRSS2 is expressed in androgen-sensitive tissues, and to some extent, the weakly significant effect of sex on disease severity in our study may reflect this proposition. The current results showed a significant association of the TMPRSS2 rs12329760 (CT + TT) SNP with increased inflammatory marker CRP, and ferritin, LDH and D-Dimer. Iwata-Yoshikawa et al. (36) used a murine TMPRSS2 knock-out to speculate that the molecule (and by implication, its genotype variants) has a role in inflammatory responses to SARS-CoV. TMPRESS2 expression in the digestive tract (37) represents a route of viral entry; thus, certain SNPs may be related to gastrointestinal symptoms of the disease such as diarrhea.

We recognise the limitation of a relatively small sample size, and so the difficulty in making further speculations regarding links between these SNPs and other research indices. Nevertheless, our data represents an advance in biomedical science because it shows the power of ACE2 (rs2285666) and TMPRSS2 (rs12329760) gene polymorphisms to predict COVID-19-related disease severity that, in addition to routine cell and serum markers, explains 86% of the severity.

## Summary Table

### What is Known About This Subject?

• SARS-CoV-2, the virus causing COVID-19, largely gains entry to the cell via the effects of ACE2 and TMPRSS2.
• Mortality is linked to co-morbidities such as age, obesity, hypertension, and diabetes, and to laboratory markers such as D-dimers, ferritin, and CRP.
• Although the greater part of our knowledge of COVID-19 has come from studies on Chinese populations, studies on other racial and ethnic groups are being described, with some discrepancies.

### What Does This Report Add?

• The leading predictors of the severity of a COVID-19 infection are hypertension, raised CRP, D-dimers and ferritin, and low platelets and lymphocytes.
• ACE2 rs2285666 and TMPRSS2 rs12329760 genotypes and alleles are linked to disease severity.
• Together, these SNPs and laboratory indices explain a large proportion of the variability in the severity of an infection.

## Data Availability

The original contributions presented in the study are included in the article/supplementary material, further inquiries can be directed to the corresponding author.

## References

[1] Ghafouri-Fard S Noroozi R Vafaee R Branicki W Poṡpiech E Pyrc K. Effects of Host Genetic Variations on Response to, Susceptibility and Severity of Respiratory Infections. Biomed Pharmacother (2020) 128:110296. 10.1016/j.biopha.2020.110296 32480226 PMC7258806

[2] Mollica V Rizzo A Massari F. The Pivotal Role of TMPRSS2 in Coronavirus Disease 2019 and Prostate Cancer. Future Oncol (2020) 16:2029–33. 10.2217/fon-2020-0571 32658591 PMC7359420

[3] Ashour HM Elkhatib WF Rahman MM Elshabrawy HA. Insights into the Recent 2019 Novel Coronavirus (SARS-CoV-2) in Light of Past Human Coronavirus Outbreaks. Pathogens (2020) 9(3):186. 10.3390/pathogens9030186 PMC7157630 32143502

[4] Dong Y Mo X Hu Y Qi X Jiang F Jiang Z. Epidemiology of COVID-19 Among Children in China. Pediatrics (2020) 145:145. 10.1542/peds.2020-0702 32179660

[5] Lek M Karczewski KJ Minikel EV Samocha KE Banks E Fennell T. Analysis of Protein-Coding Genetic Variation in 60,706 Humans. Nature (2016) 536(7616):285–91. 10.1038/nature19057 27535533 PMC5018207

[6] Hoffmann M Kleine-Weber H Schroeder S Krüger N Herrler T Erichsen S. SARS-CoV-2 Cell Entry Depends on ACE2 and TMPRSS2 and Is Blocked by a Clinically Proven Protease Inhibitor. cell (2020) 181:271–80. e278. 10.1016/j.cell.2020.02.052 32142651 PMC7102627

[7] Oladejo BO Adeboboye CF Adebolu TT. Understanding the Genetic Determinant of Severity in Viral Diseases: a Case of SARS-Cov-2 Infection. Egypt J Med Hum Genet (2020) 21:1–11. 10.1186/s43042-020-00122-z PMC7773422 38624552

[8] Kuba K Imai Y Penninger J. Angiotensin-converting Enzyme 2 in Lung Diseases. Curr Opin Pharmacol (2006) 6:271–6. 10.1016/j.coph.2006.03.001 16581295 PMC7106490

[9] Lucas JM Heinlein C Kim T Hernandez SA Malik MS True LD. The Androgen-Regulated Protease TMPRSS2 Activates a Proteolytic cascade Involving Components of the Tumor Microenvironment and Promotes Prostate Cancer Metastasis. Cancer Discov (2014) 4:1310–25. 10.1158/2159-8290.cd-13-1010 25122198 PMC4409786

[10] Stopsack KH Mucci LA Antonarakis ES Nelson PS Kantoff PW. TMPRSS2 and COVID-19: Serendipity or Opportunity for Intervention? Cancer Discov (2020) 10:779–82. 10.1158/2159-8290.cd-20-0451 32276929 PMC7437472

[11] Heurich A Hofmann-Winkler H Gierer S Liepold T Jahn O Pohlmann S. TMPRSS2 and ADAM17 Cleave ACE2 Differentially and Only Proteolysis by TMPRSS2 Augments Entry Driven by the Severe Acute Respiratory Syndrome Coronavirus Spike Protein. J Virol (2014) 88:1293–307. 10.1128/jvi.02202-13 24227843 PMC3911672

[12] Singh H Choudhari R Nema V Khan AA. ACE2 and TMPRSS2 Polymorphisms in Various Diseases with Special Reference to its Impact on COVID-19 Disease. Microb Pathogenesis (2021) 150:104621. 10.1016/j.micpath.2020.104621 PMC7709597 33278516

[13] Salvamani S Tan H Thang W Ter H Wan M Gunasekaran B. Understanding the Dynamics of COVID-19; Implications for Therapeutic Intervention, Vaccine Development and Movement Control. Br J Biomed Sci (2020) 77:168–84. 10.1080/09674845.2020.1826136 32942955

[14] Torretta S Zuccotti G Cristofaro V Ettori J Solimeno L Battilocchi L. Diagnosis of SARS-CoV-2 by RT-PCR Using Different Sample Sources: Review of the Literature. Ear Nose Throat J (2020) 100(2_Suppl. l):131S–138S. 10.1177/0145561320953231 32865458 PMC7459180

[15] Bai HX Hsieh B Xiong Z Halsey K Choi JW Tran TML. Performance of Radiologists in Differentiating COVID-19 from Non-COVID-19 Viral Pneumonia at Chest CT. Radiology (2020) 296:E46–E54. 10.1148/radiol.2020200823 32155105 PMC7233414

[16] Lindahl TL Lundahl TH Fransson SG. Evaluation of an Automated Micro-latex D-Dimer Assay (Tina-quant on Hitachi 911 Analyser) in Symptomatic Outpatients with Suspected DVT. Thromb Haemost (1999) 82:1772–3. 10613672

[17] Yan X Landen S Jacques M Papadimitriou I. PL-030 the Effects of ACE Gene Polymorphisms on ACE Content before and after High-Intensity Interval Exercise. Exerc Biochem Rev (2018) 1(1):1. 10.14428/ebr.v1i1.8483

[18] Chaudhary M. COVID-19 Susceptibility: Potential of ACE2 Polymorphisms. Egypt J Med Hum Genet (2020) 21:1–8. 10.1186/s43042-020-00099-9 PMC7502288 38624559

[19] South AM Shaltout HA Washburn LK Hendricks AS Diz DI Chappell MC. Fetal Programming and the Angiotensin-(1-7) axis: a Review of the Experimental and Clinical Data. Clin Sci (2019) 133:55–74. 10.1042/cs20171550 PMC6716381 30622158

[20] Verdecchia P Cavallini C Spanevello A Angeli F. The Pivotal Link between ACE2 Deficiency and SARS-CoV-2 Infection. Eur J Intern Med (2020) 76:14–20. 10.1016/j.ejim.2020.04.037 32336612 PMC7167588

[21] Zhao Q Hixson JE Rao DC Gu D Jaquish CE Rice T. Genetic Variants in the Apelin System and Blood Pressure Responses to Dietary Sodium Interventions: a Family-Based Association Study. J Hypertens (2010) 28:756–63. 10.1097/hjh.0b013e3283370d32 20125035 PMC2905479

[22] Zhong J Yan Z Liu D Ni Y Zhao Z Zhu S. Association of Angiotensin-Converting Enzyme 2 Gene A/G Polymorphism and Elevated Blood Pressure in Chinese Patients with Metabolic Syndrome. J Lab Clin Med (2006) 147:91–5. 10.1016/j.lab.2005.10.001 16459167 PMC7127450

[23] Benjafield A Wang WY Morris BJ. No Association of Angiotensin-Converting Enzyme 2 Gene (ACE2) Polymorphisms with Essential Hypertension*1. Am J Hypertens (2004) 17:624–8. 10.1016/j.amjhyper.2004.02.022 15233982 PMC7110370

[24] Patnaik M Pati P Swain SN Mohapatra MK Dwibedi B Kar SK. Association of Angiotensin-Converting Enzyme and Angiotensin-Converting Enzyme-2 Gene Polymorphisms with Essential Hypertension in the Population of Odisha, India. Ann Hum Biol (2014) 41:145–52. 10.3109/03014460.2013.837195 24112034

[25] Lu N Yang Y Wang Y Liu Y Fu G Chen D. ACE2 Gene Polymorphism and Essential Hypertension: an Updated Meta-Analysis Involving 11,051 Subjects. Mol Biol Rep (2012) 39:6581–9. 10.1007/s11033-012-1487-1 22297693

[26] Yi L Gu Y Wang X An L Xie X Shao W. Association of ACE, ACE2 and UTS2 Polymorphisms with Essential Hypertension in Han and Dongxiang Populations from north-western China. J Int Med Res (2006) 34:272–83. 10.1177/147323000603400306 16866021

[27] Fan X-h. Wang Y-b. Wang H Sun K Zhang W-l. Song X-d. Polymorphisms of Angiotensin-Converting Enzyme (ACE) and ACE2 Are Not Associated with Orthostatic Blood Pressure Dysregulation in Hypertensive Patients. Acta Pharmacol Sin (2009) 30:1237–44. 10.1038/aps.2009.110 19684612 PMC4007186

[28] Wu Y-H Li J-Y Wang C Zhang L-M Qiao H. TheACE2G8790A Polymorphism: Involvement in Type 2 Diabetes Mellitus Combined with Cerebral Stroke. J Clin Lab Anal (2017) 31:e22033. 10.1002/jcla.22033 PMC6817163 27500554

[29] Luo Y Liu C Guan T Li Y Lai Y Li F. Association of ACE2 Genetic Polymorphisms with Hypertension-Related Target Organ Damages in South Xinjiang. Hypertens Res (2019) 42:681–9. 10.1038/s41440-018-0166-6 30542083 PMC6477792

[30] Srivastava A Bandopadhyay A Das D Pandey RK Singh V Khanam N. Genetic Association of ACE2 Rs2285666 Polymorphism with COVID-19 Spatial Distribution in India. Front Genet (2020) 11:564741. 10.3389/fgene.2020.564741 33101387 PMC7545580

[31] Xu P Zhou Q Xu J. Mechanism of Thrombocytopenia in COVID-19 Patients. Ann Hematol (2020) 99:1205–8. 10.1007/s00277-020-04019-0 32296910 PMC7156897

[32] Badawi A. Hypercytokinemia and Pathogen-Host Interaction in COVID-19. Jir (2020) 13:255–61. 10.2147/jir.s259096 PMC7320995 32606886

[33] Caricchio R Gallucci M Dass C Zhang X Gallucci S Fleece D. Preliminary Predictive Criteria for COVID-19 Cytokine Storm. Ann Rheum Dis (2021) 80:88–95. 10.1136/annrheumdis-2020-218323 32978237

[34] Strope JD PharmDTMPRSS2 CHC Figg WD. TMPRSS2: Potential Biomarker for COVID-19 Outcomes. J Clin Pharmacol (2020) 60:801–7. 10.1002/jcph.1641 32437018 PMC7280622

[35] Singh H Choudhari R Nema V Khan AA. ACE2 and TMPRSS2 Polymorphisms in Various Diseases with Special Reference to its Impact on COVID-19 Disease. Microb Pathog (2020) 150:104621. 10.1016/j.micpath.2020.104621 33278516 PMC7709597

[36] Iwata-Yoshikawa N Okamura T Shimizu Y Hasegawa H Takeda M Nagata N. TMPRSS2 Contributes to Virus Spread and Immunopathology in the Airways of Murine Models after Coronavirus Infection. J Virol (2019) 93:1. 10.1128/JVI.01815-18 PMC6401451 30626688

[37] Bertram S Heurich A Lavender H Gierer S Danisch S Perin P. Influenza and SARS-Coronavirus Activating Proteases TMPRSS2 and HAT Are Expressed at Multiple Sites in Human Respiratory and Gastrointestinal Tracts. PloS one (2012) 7:e35876. 10.1371/journal.pone.0035876 22558251 PMC3340400

